# Role of NF-κB in Ageing and Age-Related Diseases: Lessons from Genetically Modified Mouse Models

**DOI:** 10.3390/cells10081906

**Published:** 2021-07-27

**Authors:** Verónica A. García-García, Josefa P. Alameda, Angustias Page, María Llanos Casanova

**Affiliations:** 1 Molecular and Translational Oncology Unit, Centro de Investigaciones Energéticas, Medioambientales y Tecnológicas (CIEMAT), 28040 Madrid, Spain; veronicaarantxa@externos.ciemat.es (V.A.G.-G.); pilar.alameda@ciemat.es (J.P.A.); a.page@ciemat.es (A.P.); 2 Biomedical Research Institute I+12, 12 de Octubre University Hospital, 28040 Madrid, Spain; 3 Centro de Investigación Biomédica en Red de Cáncer (CIBERONC), 28029 Madrid, Spain

**Keywords:** ageing, age-related diseases, cancer, NF-κB, transgenic mouse models of NF-κB

## Abstract

Ageing is a complex process, induced by multifaceted interaction of genetic, epigenetic, and environmental factors. It is manifested by a decline in the physiological functions of organisms and associated to the development of age-related chronic diseases and cancer development. It is considered that ageing follows a strictly-regulated program, in which some signaling pathways critically contribute to the establishment and maintenance of the aged state. Chronic inflammation is a major mechanism that promotes the biological ageing process and comorbidity, with the transcription factor NF-κB (nuclear factor kappa-light-chain-enhancer of activated B cells) as a crucial mediator of inflammatory responses. This, together with the finding that the activation or inhibition of NF-κB can induce or reverse respectively the main features of aged organisms, has brought it under consideration as a key transcription factor that acts as a driver of ageing. In this review, we focused on the data obtained entirely through the generation of knockout and transgenic mouse models of either protein involved in the NF-κB signaling pathway that have provided relevant information about the intricate processes or molecular mechanisms that control ageing. We have reviewed the relationship of NF-κB and premature ageing; the development of cancer associated with ageing and the implication of NF-κB activation in the development of age-related diseases, some of which greatly increase the risk of developing cancer.

## 1. Introduction

Ageing is considered a multifactorial and complex process, which includes genetic, epigenetic, and environmental aspects [1], and that appear as a consequence of the action of time on living beings. It is characterized by a progressive detriment of physiological functions, loss of homeostasis, and tissue and organ dysfunction, which implies the decrease in the ability of adaptation of organisms to environmental changes as well as the capability to respond to damaging agents. As a consequence, vulnerability increases and sub-lethal lesions accumulate, increasing the risk of morbidity, and eventually leading to death [2]. 

It has been proposed that the different factors that either promote premature ageing or delay this process converge in the signaling through the NF-κB pathway [3], which can be explained by the special characteristics of this protein complex that is found in almost all animal cell types, and is involved in cellular responses to different stimuli, such as genotoxic stress, free radicals, ultraviolet irradiation, and inflammation. In particular, NF-κB is known as a fundamental mediator of inflammatory responses that regulates multiple aspects of the innate and adaptive immunity [4]. This function of NF-κB is crucial for ageing, since one of the major changes that occur during the ageing process is the dysregulation of the immune response, which leads to a chronic systemic inflammatory state, usually observed in age-related diseases [5]. Therefore, its preponderant role in inflammation, together with the previous findings showing that pro-ageing stimuli activate NF-κB signaling while those with an anti-ageing effect inhibit it and, more importantly, that the inhibition of NF-κB activity can delay and even reverse ageing manifestations in human and mouse models of ageing, has made NF-κB a key protein complex that acts as a driver for ageing and to be considered as a potential therapeutic target to prevent premature ageing and age-related diseases, including cancer [3,6,7]. 

The best model to analyze the molecular and cellular basis of ageing and cancer is an organism. Accordingly, the use of mouse models in ageing research has provided essential information about the hallmarks of human ageing as well as the main signaling pathways involved in the control of this process [8,9]. Therefore, herein we will review what has been learned through the study of mouse models of components of the NF-κB pathway about the relationship of NF-κB with ageing, with age-related diseases, and with the development of cancer as a consequence of ageing. 

## 2. The Transcription Factor NF-κB

NF-κB is an important dimeric transcription factor, expressed in the cytoplasm of all cell types. It plays a crucial role in various biological processes, including immune response, inflammation, cell survival and development, response to stress, and maturation of different cell types [10]. Although activation of NF-κB is required to protect organisms from adverse environmental effects, the deregulated activity of NF-κB leads to the development of cancer and various autoimmune disorders, including rheumatoid arthritis, atherosclerosis, inflammatory bowel diseases, and multiple sclerosis.

The NF-κB signaling system consists of five members in mammals that form heterodimers or homodimers: RELA (p65), RELB, c-RER, NF-κB1 (p105/p50), and NF-κB2 (p100/p52) [11,12]. The different dimeric complexes are expressed in any cell type, p65/p50 being the predominant dimer in many tissues [13]. p65, RELB, and c-REL have a DNA-binding domain (REL homology domain), and a transactivation domain through which they activate gene transcription. In contrast, p50 and p52 only have a DNA-binding domain and lack a transactivation domain, so p50 and p52 are only capable of promoting gene transcription if they form a heterodimer with p65, RELB, and c-REL; also, it was reported that in certain circumstances, such as described in the case of *cyld*-deficient keratinocytes, BCL3 accumulates in the nucleus and activates the NF-κB subunits p50 and p52 [14], although BCL3 works more commonly by inhibiting the DNA binding and trans-activation of NF-κB heterodimer p50–p65 [15].

Two main NF-κB activation pathways have been described: the canonical or classical pathway and the non-canonical or alternative pathway. 

**Figure 1:**
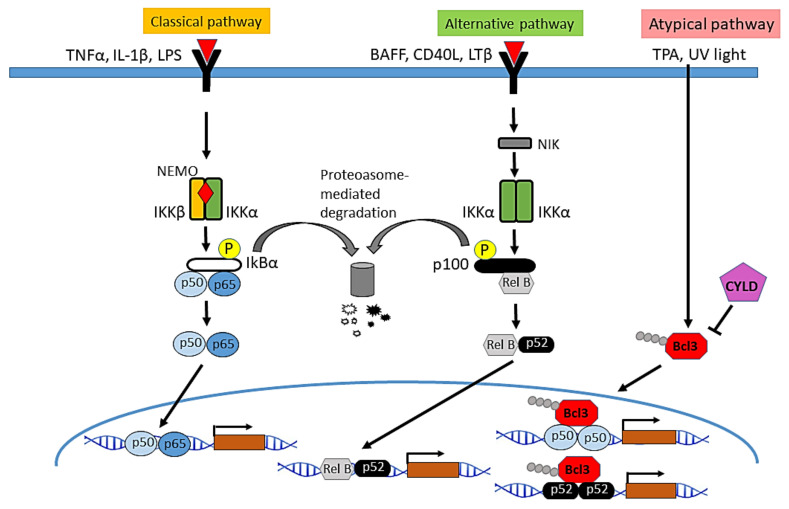
Main signaling pathways of NF-κB activation. In the classical pathway, the IKK complex is activated in response to different molecules such as the pro-inflammatory cytokines TNF-α and IL-1β, or lipopolysaccharides (LPS), followed by phosphorylation and degradation of the NF-κB inhibitor IκBα, the release of the dimers of NF-κB and their translocation into the nucleus, where they induce the transcription of multiple NF-κB target genes. The activation of NF-κB through the alternative pathway depends on IKKα homodimers. It is activated in response to cytokines such as lymphotoxin β (LTβ), the B-cell activating factor (BAFF), or the CD40 ligand. NIK kinase phosphorylates and activates IKKα leading to the processing of p100 and the formation of the heterodimer RELB/p52, which enters the nucleus and regulates the transcription of target genes. In the atypical pathway of NF-κB activation described in keratinocytes, in response to TPA or UV light, CYLD translocates from the cytoplasm to the perinuclear region where it deubiquitinates BCL3 and prevents nuclear accumulation of p50/BCL3 or p52/BCL3, thereby inhibiting the activation of NF-kB.

The classic inhibitory proteins in the NF-κB signaling system are formed by the IκB family: IκBα, IκBβ, IκBɛ, and the precursor proteins p100 and p105. In unstimulated cells, IκB binds to the nuclear localization sequence of NF-κB dimers, inhibiting their translocation to the nucleus. After cellular stimulation by pro-inflammatory signals such as cytokines (IL-1β, TNF-α), T and B cell receptors, pathogen-associated molecules, and lipopolysaccharides, the receptors of the TNF family, in this case TRAF2 and TRAF6, with ubiquitin ligase K63 function, induce the autoubiquitination and ubiquitination of target proteins involved in the activation of the IKK kinase complex, which is formed by two catalytic subunits, IKKα and IKKβ, and a regulatory subunit, IKKγ or NEMO. The activation of the IKK complex leads to phosphorylation, polyubiquitination, and degradation of IκBs, mainly of IκBα, releasing the dimers of NF-κB and allowing its translocation to the nucleus [11,16,17,18] and posterior activation of their corresponding target genes. In this pathway, IKKβ plays a central role in the activation of NF-κB in response to inflammatory stimuli [19,20]. Likewise, in spite of the lack of catalytic function of IKKγ, this protein is also essential for NF-κB activation [21]. Although IKKβ and IKKα share structural and biochemical similarities, different subcellular localization and distinct physiological and pathological roles have been described, thus, while IKKβ is predominantly cytoplasmic and plays a central role in the activation of NF-κB in the canonical pathway in response to inflammatory stimuli, the kinase activity of IKKα in the IKK complex has been found to be less efficient [22]. The IKK Complex is a central regulator of NF-κB, while IKKα has a nuclear localization signal (NLS) and shuttle between the cytoplasm and the nucleus where it develops relevant functions, such as controlling cellular proliferation and differentiation) [23,24] (Figure 1).

The activation of the non-canonical or alternative pathway of NF-κB is mediated by the participation of IKKα homodimers and the processing of p100 and p105 (which act as IκB inhibitors), and does not need the formation of the IKK complex [17,25]. Stimuli that activate non-canonical signaling include β-lymphotoxin (LTβ), B-cell activating factor (BAFF), CD40 ligand, and the receptor ligand activator NF-κB (RANKL). Binding of the activator ligand to the receptor induces the activation of NF-κB-inducing kinase (NIK), which in turn activates a complex formed by dimers of IKKα, which phosphorylates p100 leading to its partial proteolysis, and the formation of the p52 protein, which dimerizes with RELB to form the p52/RELB heterodimer [8]. This pathway is essential for the development of both lymphoid organs [26,27] and mammary gland [28]. 

In addition to the positive regulation of NF-κB by phosphorylation and subsequent ubiquitination and degradation by S6 proteasome, which allows NF-κB to translocate to the nucleus, NF-κB is negatively regulated through K63-deubiquitinases, such as A20 and CYLD: A20 interacts with TRAF1/TRAF2 and inhibits the activation of NF-κB [29]; CYLD acts on several proteins of the pathway (TRAF2, TRAF6, BCL3 and IKKγ), and negatively regulates NF-κB activation [30,31,32]. Thus, an atypical mechanism of NF-κB activation has been found in keratinocytes, in which the K63-deubiquitinase CYLD physically interacts with BCL-3—an atypical IκB protein, along with IκBζ, IκBNS and IκBη, that exhibits its function mainly in the nucleus, and is not degraded by IKK activation [33]—through a specific region that is different from the domain that mediates binding to TRAF2 and NEMO. In this atypical pathway CYLD inhibits the K63-ubiquitination of BCL-3 and its nuclear translocation. 

## 3. Role of NF-κB in Ageing

### 3.1. Pathways Related to Ageing That Converge in the Regulation of NF-κB Activation 

Different biochemical pathways have been described to influence the longevity of many animal species, from flies and worms to mice and humans. However, NF-κB appears as the central factor in which all these pathways finally converge, since those signals that promote ageing activate NF-κB and, by contrast, those that promote longevity inhibit the activation of this pathway [3]. Thus, it has been reported that survival and pro-growth pathways, known to promote ageing phenotypes, such as Insulin/IGF-1 signaling [1] stimulate NF-κB, i.e., the Insulin/IGF-1 on one hand activates PI-3K/AKT, which in turn stimulates NF-κB [34,35] and, on the other hand, activates the mammalian target of rapamycin (mTOR) [36] (a mediator of stress response which acts as a pro-ageing factor [37]), that in turn, activates NF-κB [38]. Furthermore, processes such as inflammation, stress pathways (including replicative and genotoxic stress), and DNA damage also promote age-related changes through mechanisms that end in the activation of NF-κB signaling [39,40]. In addition, the telomere shortening, a well-recognized pro-ageing event, causes genomic instability, cellular senescence, and promote ageing through the activation of NF-κB and increased levels of COX-2 and ROS (reactive oxygen species) [41]; in turn, NF-κB positively regulates protein expression levels of the catalytic subunit TERT and promotes telomere shortening [42]. As a result of these, a feedback loop of TERT with NF-κB is caused; signaling of the latter promotes macrophage polarization toward an inflammatory M1 phenotype with increased expression of IL-6 and TNF-α intensifying inflammation [43]. As an effect of the activation of NF-κB by these different pro-aging pathways, NF-κB, in turn, promotes ageing by inducing changes that contribute to cellular senescence, SASP (senescence-associated secretory phenotype), apoptotic signals, and inflammatory responses [3].

Conversely, well-known longevity factors, such as SIRT, FOXO, and caloric restriction, inhibit NF-κB signaling; i.e., SIRT1 and SIRT6 (class II histone deacetylases members of the sirtuin family, which regulates lifespan [44]), directly interact with p65, causing the repression of the transcriptional activity of NF-κB [45,46]; similarly, FOXO3A and certain variants of FOXO1 that also extend lifespan through inhibition of the activation of NF-κB, as well as caloric restriction (a mediator that improves lifespan and ameliorates age-related pathologies), which mainly exerts its beneficial effects through the inhibition of NF-κB activation and inflammation [47]. 

### 3.2. Role of NF-κB in the Maintenance and Reversion of the Ageing State

Numerous studies have shown an increase in NF-κB activity with ageing; i.e., human fibroblasts from aged individuals and from patients with Hutchinson-Gilford progeria syndrome exhibit over-activation of NF-κB [48,49]; DNA binding to NF-κB is increased in the skin, liver, kidney, cerebellum, heart muscle, and gastric mucosa of elderly mice [50,51,52]. Importantly, chronic activation of NF-κB has been observed in numerous diseases associated with ageing [3], including muscular atrophy [53], atherosclerosis [54], osteoporosis [55], heart diseases [56], type 1 and type 2 diabetes [57,58], osteoarthritis [59], and neurodegenerative diseases, such as Alzheimer’s disease and Parkinson’s disease [60,61,62,63]. In addition, it is highly relevant to note that in gene expression pattern experiments, NF-κB has been identified as the transcription factor most associated with ageing in mice [48]. 

A conclusive data was provided by the analysis of *Nfκb1^−/−^* mice deficient in both the p105 precursor and p50 NF-κB subunit, which showed constitutive activation of NF-κB (with p65/p65 NF-κB dimers) and a phenotype of premature ageing, demonstrating that hyperactivation of NF-κB was sufficient to induce accelerated ageing [64,65] (Table 1).

In agreement with this result, our group recently described the role of tumor suppressor *CYLD* as a protector from ageing in vivo, in transgenic mice; i.e., the expression of the mutant CYLD^C/S^ protein (which is functionally inactive and acts as a dominant negative) in keratinocytes and other cells expressing the keratin 5 (K5) leads to the development of a premature ageing phenotype in these K5-*CYLD*^C/S^ mice. We found that these transgenic mice presented chronic over-activation of NF-κB and accelerated ageing in numerous organs, such as skin, thymus, pancreas, and liver and, additionally, they spontaneously developed different types of tumors: skin, lung, and gastric cancer, and hepatocarcinomas [66] (Table 1). Of notice, we reported that K5-*Ikkβ* transgenic mice that overexpress IKKβ in keratinocytes and other K5-expressing cells, exhibited chronic activation of the canonical NF-κB signaling pathway, and developed spontaneous oral cancer [67]. Now, we further analyze the phenotype of these transgenic mice and observed that they have prematurely aged skin, showing epidermal atrophy along with thickened areas of the epidermis, resembling wrinkles; in addition, a strong reduction in adipose tissue and alopecia was detected, which was evident in the first months of life (unpublished results) (Table 1). 

Therefore, these results strengthen previous data of different studies linking NF-κB activation with premature ageing and cancer development [65,66,68]. However, these studies did not demonstrate a causal relationship between NF-κB activation and ageing. Retrospective studies have suggested that the maintenance of the ageing state requires active signaling programs. 

Several studies have demonstrated that the pharmacological blockade of NF-κB extends natural longevity in wild-type flies [69] and mice [70]. In addition, NF-κB inhibitory strategies have also extended the longevity of genetically modified transgenic mice [68,71,72]; and allow the reversal of many features of aging upon acute blockade of NF-κB [48], supporting the suggestion that the maintenance of the aged-related phenotypes requires an active signaling program, i.e., the permanent activation of NF-κB [73]. Examples in which the reversal of ageing after inhibition of NF-κB activation has been demonstrated include animal models carrying mutations in genes involved in ageing and representing models of progeria. Thus, in *Sirt-6*-deficient mice, the genetic inhibition of *NF-κB* ameliorated the progeroid syndrome [46] (Table 1); in murine models of *XFE-ERCC-1* (*Ercc1^−/−^ and Ercc1^−/^*^Δ^ mice) with progeroid syndrome due to defects in DNA repair, genetic reduction of an allele of *Rela*, or treatment with a pharmacological inhibitor of NF-κB activation delayed age-related symptoms and pathologies and reduced oxidative damage of DNA [74] (Table 1); in mice exhibiting a progeria syndrome, *Zmpste24^−/−^* and *LamnaG609G/G609G* mice (carrying mutations that prevented the expression or maturation of laminaA, component of the nuclear lamina), the blockade of NF-κB signaling completely prevented ageing-related skin changes [68] (Table 1). Skin changes associated with premature ageing were also reversed in the *p16INK4a* mouse model of acute radiation dermatitis as a consequence of the attenuation of the expression of NF-κB-dependent genes [75]; in *IKKβ* mice that suffer muscle wasting (which represent a model of sarcopenia, i.e., muscle loss due to ageing), NF-κB blockade reversed muscle ageing manifestations [53]; similarly, a mouse model of *DMD* (dystrophin-deficient mice) and progeria, the deletion of one allele of *Rela* or the lack of *Ikkβ*, reversed the ageing features and muscle pathology [76] (Table 1). Furthermore, it was shown that ageing retardation could be achieved via IKKβ/NF-κB inhibition across the brain [77] (Table 1). Moreover, parabiosis experiments, in which the circulatory systems of young and old mice were artificially connected, showed the potential of systemic soluble factors both to prevent several manifestations of ageing [78], and to induce pro-ageing effects [79]. In the latter case, signaling molecules secreted by old animals contributed to the development of progeria by acting on distant organs and then amplifying the cascade of ageing signals. Importantly, in this signaling pathway by soluble systemic factors, NF-κB played a key role by integrating the autonomic and systemic signals of ageing. 

## 4. Study of Age-Related Diseases through the Analysis of Genetically Modified Mouse Models of the NF-κB Signaling Pathways

The use of transgenic mice of the components of the NF-κB pathway has revealed the decisive role of this pathway in both the establishment and progression of age-associated diseases, as well as providing putative targets for the treatment of these chronic and devastating conditions. 

### 4.1. A Tight Relationship between NF-κB Activation-Inflammation- and Age-Associated Diseases

Chronic inflammation has been considered a predominant and recurrent factor that is associated with physiological and pathological ageing processes. Therefore, the group of Benedictis coined the term “inflamm-aging” to refer to the proinflammatory status of aging, which is a major characteristic of the aging process being provoked by a continuous antigenic load and stress [80,81,82], constituting the biological background that favors susceptibility to the development of age-related diseases/disabilities with an inflammatory pathogenesis, such as atherosclerosis, Alzheimer’s disease, osteoporosis, and type 2 diabetes (T2D). These authors also described that the activation of the macrophages with age (macroph-ageing) has a relevant role in the inflamm-aging. It is relevant that during aging, adaptive immunity significantly declines (immunosenescence), whereas innate immunity is activated and induces the characteristic pro-inflammatory profile of the elderly, being the master regulator of the innate immunity the NF-κB system. The activation of NF-κB induces the transcription of IL-6 and TNF-α, whose levels of expression are low or undetectable in most of the young people and start to increase in healthy people at about 50–60 years of age. Therefore, the beneficial effect of the defense system network (innate immunity, stress, and inflammation), dedicated to the neutralization of dangerous/harmful agents early in life and in adulthood, turns out to be detrimental late in life, since increased levels of chronic inflammation induces destructive processes inside cells, rapidly producing more NF-κB activation and an accelerating cycle of inflammation. This results in cell death, tissue loss, DNA damage, and other harmful changes and diseases that come with aging.

NF-κB is the key transcription factor found on the axis of the ageing inflammatory network: it is activated by different factors, such as mitochondrial dysfunction, oxidative stress, activation of innate/inflammatory responses, induction of the Insulin/IGF-1 pathway, and DNA damage [83]. All of these events increase with age and determine a sustained activation of NF-κB, which induces a host defense mechanism, responsible for the release of a series of molecules known as SASP. SASP occurs in various cell types (fibroblasts, epithelial and endothelial cells, astrocytes, preadipocytes, and leukocytes, among others) and is characterized by an increase in the secretion of approximately 40–80 factors involved in different intracellular signaling pathways [84]; they comprise soluble proteins (interleukins, chemokines, growth factors, and proteases) and insoluble or non-protein components such as ROS. Likewise, SASP collaborates in the chronic inflammation observed in ageing, favoring in turn the ageing process and the appearance of degenerative diseases related to advanced age (Alzheimer’s, Parkinson’s, atherosclerosis, arthritis, osteoporosis, sarcopenia, type II diabetes, dry eye, disease, etc.) [83]. Consequently, an elevated activation of NF-κB has been related to age-associated diseases that course with inflammation [3,11,85], wherein, in addition, and given the crucial role of NF-κB in other diverse biological processes, including immune response, macrophage polarization toward an inflammatory M1 phenotype, telomere shortening, cellular senescence, response to stress etc. [10], other functions of NF-κB besides to the inflammatory one may be linked to the development of a specific age-related disease, although this function ultimately results in a greater activation of NF-κB and an increases in the inflammation. Accordingly, we mentioned in Table 2, Table 3, Table 4 and Table 5 (“proposed mechanisms” column) the involvement of these additional functions of NF-κB in the development of a specific age-related disease, as described by the authors. 

Likewise, in addition to the inflammatory background that indicates the necessary basis for the development of age-related diseases and disabilities (such as atherosclerosis, Alzheimer’s dementia, osteoarthritis, type 2 diabetes, etc.), the genetic characteristics of each individual combined with environmental (stress) ones are factors that can also contribute to the susceptibility of each individual to develop a specific age-associated disease.

### 4.2. Involvement of NF-κB in Age-Associated Eye Diseases 

There are several eye conditions whose main risk factor is increasing age, among them are glaucoma, cataracts, dry eye diseases (DED), and macular degeneration. Most of these diseases are related to the activation of NF-κB in different eye components. For the study of the molecular mechanisms and symptoms of eye diseases, mouse strains carrying genetic anomalies similar to those affecting humans have often been analyzed (such as in age-related macular degeneration studies [86]). Herein, we show how the use of mouse models of the NF-κB pathways has also contributed to the knowledge of the molecular bases of these frequent diseases in the ageing population. For example, they have been used in studies of glaucoma. This is a disorder that comprises a group of eye diseases characterized by chronic degeneration of retinal ganglion cell (RGC) axons in the optic nerve, death of RGC somas in the retina, and loss of synapses in the retina and brain. Glaucoma may cause irreversible blindness, since the optic nerve results damaged. Among causative factors of this disease, neuroinflammation has been considered an important factor that plays a relevant role as a consequence of reactive glia, and, the use of transgenic mice with a conditional ablation of *Ikkβ* in astroglia unveils the key role of NF-κB in neuroinflammatory and neurodegenerative outcomes of glaucoma [87] (Table 2). 

In addition, transgenic mice of the NF-κB pathway were used in studies about DED. This is a disorder that mainly affects people over 50, primarily postmenopausal women, and can result from not enough tear production, poor quality of tears causing evaporation from the eye surface, or a combination of both. Complications that scar the surface of the eye, leading to reduced vision, may appear. It is widely recognized that inflammation has a significant role in the etiopathogenesis of DED [88], and the use of mouse models of NF-κB has allowed to demonstrate that chronic keratitis (DED) is independent of TNFR1 signaling but dependent on NF-κB [89] (Table 2). 

### 4.3. Role of NF-κB in Age-Related Neurological Diseases

NF-κB is involved in the pathogenesis of a variety of neurodegenerative disorders, including Parkinson’s disease (PD), Alzheimer’s disease (AD), Huntington’s disease (HD), diabetic neuropathy, and amyotrophic lateral sclerosis (ALS) [90,91]. The development of several of these pathologies (PD, AD, ALS) is associated to ageing. 

The analysis of transgenic mice with the NF-κB signaling pathway altered has provided significant information about the origin and progression of these disorders. 

Parkinson’s disease usually begins in middle or advanced stages of life and the risk of development of this disorder increases with age, 60-year olds and older individuals being the most susceptible. PD is the most common movement disorder, characterized by abnormal α-synuclein deposition in fibrillary aggregates composing intraneuronal inclusions in different brain areas, such as the substantia nigra, olfactory bulbs, hypothalamus, and cranial nerve motor nuclei; the proteinaceous inclusions of α-synuclein are key neuropathological hallmarks of the brain of affected patients, and, in addition to motor symptoms, PD patients can also suffer from other non-motor pathologies [92]. The molecular causes of Parkinson´s diseases remain to be fully elucidated. Different findings point towards a role of p65 in PD, i.e., nuclear levels of p65 are highly increased in nigral dopamine (DA) neurons and glial cells of PD patients; and inhibition of p65 exerts neuroprotection against neural toxicity, suggesting that p65 upregulation may play a role in dopaminergic neuron degeneration [61]. However, in vivo results in knockout mice deficient in *c-Rel* showed that it can exert neuroprotective actions [90,92,93]. Therefore, it seems that there is a possible interplay between NF-κB dysregulation and α-synuclein deposition in the pathology of PD; i.e., as observed in *c-Rel*-deficient mice, by affecting key molecular pathways, disturbances in the activation of NF-κB factors may contribute to PD pathogenesis by favoring α-synuclein accumulation and aggregation, leading to glial cell activation and neuronal cell death [92,93]. These same processes may increase NF-κB activation, initiating a vicious circle perpetrating disease progression. Here, we illustrate examples of the use of *c-Rel* knockout mice to study the development of PD symptoms in aged mice (Table 2).

AD appears more often in people over 65 years-old. It is the most common form of dementia and a progressive, chronic neurodegenerative disorder characterized clinically by a deep deficit in the ability to form new memories, causing problems in thinking, language, planning, and behavior [94,95]. AD neuropathology includes the formation of extracellular plaques and intracellular neurofibrillary tangles (NFTs). NFTs occur in brain tissues as a result of both Amyloidβ (Aβ) agglomeration and Tau phosphorylation. 

Activation of NF-κB has been detected in degenerating neurons in the brains of AD patients [96], and in neuronal and glial nuclei proximal to early stage plaques [97]. Evidence shows that NF-κB may regulate the production of the Aβ42 oligomer. This suggests the possibility that deregulated activation of NF-κB in neurons may lead to increased Aβ production and actively participate in the progression of AD. It has been described that NF-κB can affect AD pathology through increased release of neuroinflammatory cytokines by activated microglia and astrocytes; in fact, NF-κB plays an active role in the progression of AD. Thus, it seems that although the NF-κB pathway is involved in normal brain functioning, its aberrant activation triggers undesirable changes, such as neuroinflammation, activation of microglia, increase in oxidative stress, and apoptotic cell death, which ultimately can lead to homeostatic abnormalities in the brain; or in the initial stages of AD, it may drive normal neurons toward a degeneration process. Here, we exemplify how the generation of transgenic mice of the NF-κB pathway with conditional deletion of the inhibitor *IκBα* revealed the cell-specific effects of NF-κB activation in neurons and astroglia [98] (Table 2).

ALS especially affects people between the ages of 50 and 70, and is more frequently detected in 60–69-year-old men, although there are cases described in younger patients. ALS is a neuromuscular degenerative disease that originates when motor neurons gradually decrease their function and die, causing progressive muscle paralysis with a fatal prognosis [99]. Expanded hexanucleotide repeats in the *C9orf72* gene account for almost 40% of the familial cases whereas other mutated genes include *superoxide dismutase 1* (*SOD1*), TAR DNA-binding protein (*TDP-43 gene*), *fused in sarcoma* (*FUS*), and *ubiquilin-2* (*UBQLN2*) [100]. Abnormal cytoplasmic accumulations of TDP-43 are a common occurrence in degenerating neurons of the CNS in the majority of ALS cases [101]. Recently, the use of transgenic mice with neuron-specific expression of a super-repressor form of the NF-κB inhibitor (*IκBα*-SR) was reported to unveil the role of neural NF-κB activity in the pathogenesis of ALS (Table 2). As a result, it has been proven that inhibition of the NF-κB signaling pathway mitigate behavioral and pathologic changes in transgenic mouse models of ALS that express mutant forms of either *TDP-43* or *SOD1* [101].

### 4.4. Senescent Osteoarthritis and Osteoporosis

Osteoarthritis (OA) and osteoporosis are both degenerative diseases, which tend to get worse over time. Their development is directly related to ageing and are more common in women, since hormonal imbalances during menopause have direct consequences on both pathologies [102].

Osteoporosis is characterized by the loss of bone mass, i.e., demineralization of the bone, along with an increased fat in the bone marrow, which results in an increased incidence of fractures. After 60 years of age, the lifetime risk of fragility fractures due to senile osteoporosis is approximately 44–65% in women and 25–42% in men [103]. Multiple characteristic factors of old age contribute to osteoporosis, including metabolic factors, hormonal imbalance (estrogen deficiency), and mechanical changes that take place in the bone tissue itself. In addition, clinical and molecular evidence suggests that inflammation also exerts a significant influence on bone turnover, inducing osteoporosis (proinflammatory cytokines are implicated in the regulation of osteoblasts and osteoclasts); thus, elevated levels of TNFα, IL-1β, IL-6, M-CSF, GM-CSF, and prostaglandins (that contribute to bone loss) are detected in post-menopausal women, evidencing the role of estrogen activity in the regulation of cytokines. It has been found that NF-κB mediates the osteolytic function of most of these cytokines and also controls their production [104]. Therefore, both chronic inflammation and remodeling of the immune system that occur in ageing appear as important pathogenic factors for the development of osteoporosis [105].

Most of the current knowledge on the mechanisms associated with osteoporosis during ageing has been generated from studies in animal models (mainly mice). Among them, the use of transgenic mice of the NF-κB signaling pathways has shown that the inhibition of the activation of this transcription factor in differentiated osteoblasts prevents osteoporotic bone loss induced by ovariectomy (procedure used to induce estrogen deficiency) [106]; also, recently, it was reported that increased NF-κB activation in osteoprogenitors (OP) in skeletally mature mice leads to ageing-associated osteoporosis-like phenotypes, demonstrating the role of this transcription factor as a master regulator of bone remodeling [107] (Table 2). Finally, these studies highlight the potential of NF-κB as a therapeutic target in inflammatory diseases of bone and in cases of adverse bone remodeling during the natural aging process.

Among the rheumatic diseases, osteoarthritis is one of the most prevalent; increasing age and obesity are the most prominent risk factors for OA development, which is a degenerative disease characterized by the loss of cartilage, where chondrocytes experience a phenotypic shift towards hypertrophy concomitant with abnormal matrix remodeling displaying increased expression and activities of matrix-degrading enzymes, such as collagenase [108,109]. In vitro approaches have shown that CHUK/IKKα, which controls the non-canonical NF-κB pathway, plays a predominant role in chondrocyte hypertrophy and matrix remodeling, in a kinase-independent manner [110]. Importantly, in vivo studies*,* through the analysis of transgenic mice with specific deletion of *Ikkα* in cartilage, has revealed that *Ikkα* deficiency reduces collagenase activity and hypertrophy-like features in articular cartilage [111], suggesting the cell-autonomous effects of IKKα in chondrocytes, showing IKKα as a critical regulator of cartilage remodeling that controls chondrocyte phenotype and impacts cell survival, matrix homeostasis, and remodeling (Table 2). 

### 4.5. Role of NF-κB in Age-Associated Muscle Diseases: Muscle Wasting, Sarcopenia, Cachexia

Muscle wasting/atrophy is the result of different conditions, such as ageing, malnutrition, muscle disuse, and any serious illness or injury that causes adult skeletal muscle fibers to become atrophic and, at the end, lead to a loss of muscle mass. Specifically, muscle loss due to ageing is known as sarcopenia, and typically happens faster around age 75, although it affects 10% of adults who are over 50 years old. Sarcopenia is a factor of frailty and probability of falls and fractures in older adults [112]. Additionally, sarcopenia can reduce skeletal muscle insulin sensitivity and it has been reported that skeletal muscle dysfunction is involved in the development of type 2 diabetes [113]. Another condition related to muscle loss is cachexia, which is a wasting condition associated with chronic illnesses, such as cancer, primarily characterized by atrophy (wasting) of skeletal muscle that leads to pronounced weight loss and contributes significantly to morbidity and mortality, mainly in cancer patients [114]. 

Studies using mouse models of the NF-κB signaling pathway have revealed that skeletal muscle atrophy/sarcopenia is associated with a marked and sustained activation of NF-κB signaling and different works have shown that IKKβ, p50, and BCL3 are required both for NF-κB activation and muscle atrophy [53,115,116,117] (Table 1 and Table 2). In addition, in a study using both *c-Rel*-deficient mice and transgenic rats expressing the *IκBα* super-repressor, it was found that while IκBα degradation was required for both decrease in fiber size and activation of NF-κB signaling observed in muscle wasting pathologies, c-REL is not necessary for the development of these types of diseases [118] (Table 2). 

Other kinds of studies using gene transfer of a dominant negative (d.n.) *Ikkα* or *Ikkβ* into rat soleus muscles showed decreased unloading-induced NF-κB activation and inhibition of atrophy by 50% when each IκB kinase was examined independently; overexpression of d.n. *Ikkβ* plus d.n. *Ikkα* showed an additive effect on the inhibition of disuse atrophy (70%), suggesting that both kinases of the IKK complex are necessary and sufficient for muscle atrophy [119]. 

The use of mouse models with expression of the desired gene of the NF-κB pathway, specifically in skeletal muscle, has allowed to find its direct involvement in the muscle phenotype and has highlighted the negative role of NF-κB in the development of the devastating muscle atrophy. In addition, additional studies [115] have analyzed other possible contributory factors outside the fiber, and within the muscle microenvironment, finding that in tumor-bearing mice and patients with pancreatic cancer, the persistent expression of a constitutive kinase-active *Ikkβ* in *Pax7*-expressing satellite cells inhibits the myogenic program that was associated with cachexia. These authors found that serum-derived cachectic factors signal through NF-κB in myogenic progenitors to cause deregulation of *Pax7* that in turn inhibits differentiation and promotes muscle wasting in cachexia (Table 2).

Therefore, these studies in mouse models of the NF-κB pathway have allowed a significant advancement in the understanding of the role of NF-κB/IκB family members in skeletal muscle atrophy that usually accompanies ageing, as well as the main cell targets of NF-κB; additionally, they have provided new candidate NF-κB target genes and suggested the broad benefits of NF-κB blockade as possible therapeutic approaches against sarcopenia. 

### 4.6. Implication of NF-κB in Age-Related Heart Diseases 

The number of people affected by heart disease increases with age in both men and women. About four out of five people who die of coronary heart disease are 65 years of age or older [120]. Therefore, understanding the critical mechanisms underlying cardiovascular ageing and age-related arterial pathophysiological alterations is an urgent issue to prevent cardiovascular diseases in older persons. 

It is relevant that in the absence of other risk factors, ageing per se promotes the development of atherosclerosis and increases the morbidity and mortality of myocardial infarction (MI) and stroke. Evidences indicate that endothelial oxidative stress (ROS) and arterial inflammatory pathways act as potent proatherogenic stimuli that play a central role in cardiovascular ageing [120,121]. In agreement, it has been found that chronic inflammation participates in the development and progression of cardiovascular diseases (atherosclerosis, aortic valve disease, MI, heart failure (HF)) and cardiometabolic disorders (obesity, insulin resistance) [122,123]. In addition, several pro-inflammatory markers are elevated in patients with cardiomyopathies, existing a direct correlation between the prognosis and severity of disease and grade of inflammation [122]. It is remarkable that the activation of NF-κB has been found to be a relevant event in cardiovascular ageing [124]. 

Herein we exemplify how the use of transgenic mice of the NF-κB pathway has helped in the understanding of the mechanisms that influence the development of cardiovascular diseases in older people. 

#### 4.6.1. Heart Failure, and Cardiac Hypertrophy and Remodeling 

With ageing, blood vessels become less flexible, making it harder for blood to move through them easily. Fatty deposits also collect along the artery walls and slow the blood flow from the heart. These and other risk factors, such as high blood pressure, smoking, and diabetes increase risk for a heart attack. The implication of IKK/NF-κB activation has been found in heart failure, myocarditis, cardiac hypertrophy, ischemia/reperfusion damage, and myocardial infarction [125]. Moreover, a relationship between inflammation and cardiovascular risk has been established, since patients with HF present augmented levels of proinflammatory cytokines such as IL-1β, IL-6, IL-18, and TNF-α. Additionally, levels of these cytokines as a reaction to myocardial damage are considered relevant prognostic factors, showing a direct correlation with mortality rates in patients [126]. Cardiovascular therapy, i.e., statins, COX-2 inhibitors, angiotensin II receptor blockers, etc., modulates cytokines expression and inflammatory signaling supporting the notionthat inflammation has a functional role in cardiovascular diseases. Therefore, it is considered that activation of NF-κB (which induces the expression proinflammatory cytokines and ROS release) plays an important role in the pathogenesis of cardiac remodeling and HF [123,127]. Accordingly, the clinical use of benzimidazole derivatives has been reported to decrease the production of intracardiac IL-1β, IL-6, TNF-α, and nitric oxide by inhibiting the activation of NF-κB [128,129] in mouse models of HF. Numerous mouse models of the NF-κB pathway have been employed in the studies of the mechanisms involved in heart failure and cardiac hypertrophy. Here we present relevant results comprising studies of the contribution of different members of the NF-κB signaling to age-associated cardiac disorders (Table 3). 

Cardiac hypertrophy and remodeling is characterized by enlargement of the heart and an increase in myocyte cell volume that occurs in response to factors such as hemodynamic stress and acute myocardial injury. It involves macro and microstructure physiological changes in order to face wall stress; prolongation of this state may lead to arrhythmias and HF. Cardiac hypertrophy is more common in older adults, mainly in those with hypertension and diabetes [130]. 

By using mice with specific *Nfkb1*-deletion both in myocyte and non-myocyte cells, it has been found that the activation of the p50 subunit of NF-κB plays an important role in the pathogenesis of myocardial hypertrophy; it also exacerbates post-infarction remodeling and mortality and promotes myocardial inflammation [130,131,132]. Thus, it was shown that targeted disruption of *Nfkb1* ameliorated myocardial hypertrophy in response to chronic infusion of angiotensin II [130], TNF-α-induced cardiomyopathy [133], or heart damage induced by ligation of the left coronary artery [131,132] (Table 3).

Using a mouse model with a inducible expression of a constitutively active form of *Ikkβ* in cardiomyocyte, it was shown that IKK/NF-κB activation induces inflammatory cardiomyopathy and HF, which could be reversed by expression of *Iκbα* super-repressor, which inhibits NF-κB, indicating that the phenotype of mice was dependent on NF-κB activation [134] (Table 3).

Reduction of NF-κB p65 activation in transgenic mice with the myocyte-selective overexpression of the *Iκbα* super-repressor improved survival and alleviated left ventricular (LV) remodeling, associated with a diminished pro-inflammatory cytokine expression, fibrosis, and apoptosis [135]; it further demonstrated that p65 modulation of cell death in HF may occur in part from NF-κB-mediated transformation of the endoplasmic reticulum stress response from one of adaptation to one of apoptosis (Table 3). In addition, in another mouse model, *Rela* deletion reduced pressure overload–induced cardiac hypertrophy with conserved cardiac function. In this case, a mechanism involving a direct interaction between the nuclear factors of activated T cells (NFAT) and NF-κB that integrates two signaling pathways to promote cardiac hypertrophy and LV remodeling [136] was suggested (Table 3). 

Overall, as a result of these data, it has been proposed that blockade of NF-κB might be a promising therapeutic strategy to attenuate cardiac remodeling and failure after myocardial infarction.

#### 4.6.2. Atherosclerosis

Atherosclerosis is a progressive disease with a strong inflammatory component, characterized by accumulation of lipids in the arterial vessel wall. Disease progression leads to build-up of atherosclerotic plaques that cause narrowing of the arterial lumen. Atherosclerotic plaques often remain stable for years, but can rapidly become unstable, and rupture and trigger thrombus formation. Accordingly, in addition to restriction of the vessel lumen, the presence of atherosclerotic plaques is linked to an increased risk of acute cardiovascular events such as myocardial infarction and stroke. In general, atherosclerosis can be considered a multi-step process with four main phases: endothelium injury, foam cell formation, smooth muscle cell proliferation, and formation and rupture of the atherosclerotic plaque. 

The use of animal models of atherosclerosis has been an essential tool to improve the understanding of the molecular mechanisms behind atherosclerotic plaque formation and progression. In general, mouse models are based on accelerated plaque formation due to a cholesterol-rich/Western-type diet, or manipulation of genes involved in the cholesterol metabolism, such as *Apolipoprotein E* deficient (*ApoE^−/−^*) and *LDL-receptor* (*LDLr*) knockout mice [137]; also, models of chronic intermittent hypoxia (CIH)-induced atherosclerosis, and the introduction of additional risk factors for atherosclerosis, such as diabetes are used. In addition, as we exemplify here, mouse models carrying changes in NF-κB activation were also generated [138]. Models of transgenic mouse deficient in *Nfkb1* gene were generated to determine the role of p50 in atherosclerosis; these studies employed mouse models of CIH-induced atherosclerosis, which were also deficient in the *Nfkb1* gene [139,140] (Table 3) (the activation of NF-κB by CIH had been first demonstrated in vitro [141]). CIH causes atherosclerosis in mice fed with a high cholesterol diet (HCD) [139] and it is believed to contribute to obstructive sleep apnea (OSA)-associated cardiovascular disorders [142], which is turn is considered as a risk factor for atherosclerotic cardiovascular disease [143]; i.e., CIH exposure causes alterations such as hypercholesterolemia, dyslipidemia, systemic and vascular inflammation, hypertension and impaired triglyceride clearance, all of which contribute to atherosclerosis development. These studies conclude that p50 protects against CIH-induced atherosclerosis by inhibiting vascular inflammation and hypercholesterolemia and that NF-κB signaling might be a central common pathway through which CIH activates multiple atherogenic mechanisms that act synergistically to induce atherosclerosis (Table 3). Additionally, it was demonstrated that the ablation of *Nemo* or the expression of a dominant negative form of *Iκbα* (which impeded NF-κB activation) specifically in endothelial cells had an atheroprotective effect, i.e., they demonstrated that endothelial NF-κB signaling orchestrates proinflammatory gene expression at the arterial wall and promotes the pathogenesis of atherosclerosis [138] (Table 3). The beneficial effect of the deficiency of *Ikkβ* in smooth muscle cells (SMCs) was also described, since the lack of this subunit of the IKK complex renders mouse models of atherosclerosis resistant to atherosclerosis by high-fat feeding [144] (Table 3). 

Therefore, all these findings identify endothelial NF-κB as a potentially important drug target for developing therapies against atherosclerosis, since targeting the NF-κB pathway can simultaneously inhibit multiple atherogenic mechanisms. 

### 4.7. Role of NF-κB Activation in the Development of Age-Associated Diseases with High Risk of Cancer Development

Extensive studies on the relationship between ageing and NF-κB activation have allowed progression in the study of the molecular basis of cancer, since they have revealed that both ageing and cancer have many molecular connections. In addition, there are many aged-related diseases that involve an enhanced risk to develop different types of cancer. 

It is relevant that in most of these disorders, the activation of NF-κB has been proven to develop a decisive role in both the establishment and progression of the disease. Among the disorders that carry a high risk of developing cancer, obesity, insulin resistance, type II diabetes, and liver diseases are highlighted.

Here we have reviewed those advances in the understanding of the cause and progression of these diseases that have been acquired through the use of transgenic mouse models of the NF-κB pathway, which recapitulate key aspects of each human condition. 

#### 4.7.1. NF-κB in Cardiometabolic Disorders: Obesity, Insulin Resistance, Type II Diabetes Mellitus

The prevalence of type 2 diabetes mellitus results from insulin resistance and consequent impaired β-cell function and survival; it is characterized by peripheral insulin resistance, increased hepatic glucose production, and impaired insulin secretion. T2D has increased considerably in recent years, reaching epidemic proportions. Also, insulin resistance associated with obesity is an important contributor to T2D development. Obese individuals exhibit elevated levels of proinflammatory cytokines, including tumor necrosis factor-α (TNF-α), interleukin-1β (IL-1β), and IL-6, giving rise to the hypothesis that obesity-induced insulin resistance is an inflammatory condition; in fact, a link between these diseases (obesity, insulin resistance and T2D) and inflammation has been established [145].

On the other hand, it has been described that excess body weight is associated with an increased risk for a range of malignancies and that the triad of obesity, insulin resistance, and adipokine aberrations is linked to cancer: there is evidence that obesity is associated with an increased risk for cancer of at least 13 anatomic sites, including endometrial, esophageal, renal, and pancreatic adenocarcinomas; hepatocellular carcinoma; gastric cardia cancer; meningioma; multiple myeloma; colorectal, postmenopausal breast, ovarian, gallbladder, and thyroid cancers [146]. 

Here we show how the use of genetically modified mouse models of the NF-κB signaling pathway has helped considerably to identify the mechanisms leading to these frequent cardiometabolic age-related disorders. Studies using *Ikkβ^+/−^* mice [147] or conditional transgenic mice of *Ikkβ* specifically in the liver [148,149] (Table 4), showed that deficiency of IKKβ protected against the development of insulin resistance during high-fat feeding. They described that IKKβ led to inhibition of insulin sensitivity through induction of proinflammatory mediators ([148,149]. Later, by using hemizygous *Rela^+/−^* mice in the liver, a new molecular mechanism for inflammation in the pathogenesis of insulin resistance was reported in which NF-κB promoted cAMP signaling activity through downregulation of *PDE3B* transcription in hepatocytes [150] (Table 4). 

The use of mouse models lacking *Ikkβ* specifically in smooth muscle cells (SMCs) [144] has allowed to find that *Ikkβ*-deficient mice were resistant to diet-induced obesity and metabolic disorders and. Additionally, the use of these transgenic mice have also revealed that IKKβ functions in SMCs to promote vascular inflammatory responses and atherosclerosis development, establishing a link between IKKβ activation-vascular inflammation-obesity (adipose tissue development) and atherosclerosis (see Table 3 and Table 4). 

The generation of transgenic mice that express the *IκBα* superrepressor specifically in endothelial cells indicated that mice lacking NF-κB activation in endothelium were protected from the development of insulin resistance associated with diet-or-genetically-induced obesity. Additionally, this study highlighted the relevant role that endothelium plays in obesity and age-related disorders through intracellular NF-κB signaling, thereby ultimately affecting life span [151] (Table 4). 

Global *NFkb1* inactivation in mice was reported to generate several metabolic changes, including resistance to diet-induced obesity and improvement in insulin resistance, with an elevation in energy expenditure [152,153,154,155,156] (Table 4). 

Therefore, all these approaches that study the role of different components of NF-κB signaling in T2D, obesity, and insulin resistance conclude that inhibition of the activation of this pathway represses insulin resistance in obesity and T2D and therefore has a beneficial effect on the symptoms of these age-associated diseases.

#### 4.7.2. Age-Related Liver Diseases

The development of the chronic inflammatory liver disease known as non-alcoholic steatohepatitis (NASH) affects people who drink little or no alcohol and is more frequent in older people. It goes along with hepatic steatosis (a buildup of fat in the liver), and can progress to cirrhosis and hepatocellular carcinoma (HCC). Although the majority of HCC develops on the background of chronic hepatitis or cirrhosis, there is also non-cirrhotic HCC. The histopathological changes associated with chronic NASH include persistent tissue infiltration by lymphocytes and neutrophils along with hepatocyte death and activation of resident liver macrophages Kupffer cells. This environment stimulates compensatory hepatocyte proliferation, a response that maintains liver mass but may also be the main driver of hepatocarcinogenesis [157]. This suggests the possibility of a mechanistic link between inflammatory mediators such as interleukin IL-6 and HCC, in which inflammation promotes hepatocarcinogenesis through production of cytokines that stimulate compensatory proliferation [158,159] and with NF-κB as the main mediator of inflammation and a relevant role in liver regeneration. 

Here, we show how the use of mouse models of different components of the NF-κB pathway (mainly *Nf-κb1*, *Rela*, *Nik*, *Ikkα*) have provided clues to the mechanisms that lead to age-related chronic liver diseases (CLD) and its progression. Thus, the use of *Nfkb1*-deficient mice has established the role of p50 in the development of ageing associated chronic liver disease (CLD), with hepatocyte telomere damage, dysplasia, and HCC development [65,160] (Table 1 and Table 4). This is because while the classic pro-inflammatory NF-κB is the p65:p50 heterodimer, the homodimer p50:p50 is an active repressor of pro-inflammatory gene transcription; thus, in the absence of p50, p65 is able to form other type of heterodimers or p65:p65 homodimers, enhancing responses to inflammatory stimuli (*Nfkb1*-null mice exhibit an increase in NF-κB activity), but without the anti-inflammatory function of p50:p50 homodimers. This results in a progressive low-grade inflammation and consequently a progeria phenotype of *Nfkb1*-deficient mice (Table 1) with numerous age-associated diseases, including CLD and HCC development [64,65] (Table 1). Later, it was reported that while NF-κB-p50 promotes lipid accumulation in hepatocytes in the progression of hepatic steatosis, through an impact in histone deacetylase 1 (HDAC1) activity, the p65 subunit did not play a direct role in the control of hepatic steatosis [156] (Table 4). However, the results obtained by our group suggest the role of p65 in the development of chronic liver diseases, i.e., the analysis of transgenic mice expressing the mutant *CYLD^C/S^* that leads to the sustained activation of NF-κB, with increased p65 signaling, showed that these mice developed a phenotype characterized by premature ageing and systemic inflammation (Table 1), which led to chronic hepatitis and the formation of HCC [66] (Table 4). Among the changes that occur in CLD, impaired reparative hepatocyte replication has been described, which contributes to disease progression [161,162,163,164]. The study of hepatocyte-specific *Nik* and *Ikkα* knockout mice has unveiled the role as suppressors of hepatocyte replication that both NIK and IKKα proteins play [165] (Table 4). In agreement with these results, it was described that hepatic NIK was aberrantly activated in both mice and humans with NAFLD or alcoholic liver disease [166]. 

Therefore, these results obtained in mouse models of the NF-κB pathway confirm the key role of different components of the NF-κB complex in the development of chronic liver diseases and HCC.

**Figure 2:**
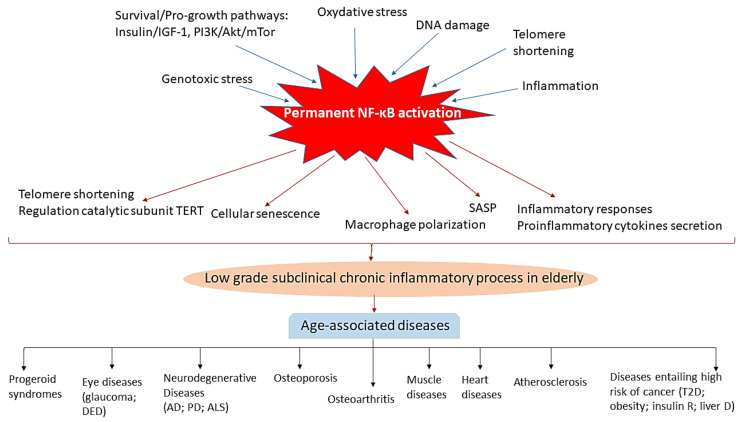
Schematic illustration highlighting the central role of chronic NF-κB activation in inflammation in the elderly and in the development of age-related diseases. Factors known to promote aging phenotypes, such as pro-growth and survival pathways, genotoxic and oxidative stress, DNA damage, telomere shortening and inflammation activate NF-κB. NF-κB in turn acts to promote ageing-related changes by contributing to telomere shortening and positive regulation of the catalytic subunit TERT, cellular senescence, macrophage polarization toward an inflammatory M1 phenotype, proinflammatory secretion of cytokines, mainly TNFα and IL6, and inflammatory responses, which originate a subclinical chronic inflammatory status in aged individuals which can lead to the development of diverse age-associated diseases, including shortening of life-span, progeroid syndromes; eye diseases (glaucoma, DED (dry eye disease etc.), neurodegenerative diseases (AD, Alzheimer’s disease; PD (Parkinson’s disease); ALS (amyotrophic lateral sclerosis) etc.), osteoporosis, osteoarthritis, muscle and heart diseases, atherosclerosis and other disorders such as type 2 diabetes (T2D), obesity and insulin resistant, and hepatic diseases, which have associated an increased risk of developing cancer.

In Figure 2, we have summarized the relationship of NF-κB with other pro-aging pathways and with the development of age-associated diseases reviewed in this manuscript.

## 5. Role of NF-κB in the Decline of Stem Cell Functions in Aged Individuals 

In addition to the age-associated diseases reviewed above, age-associated anatomic and functional changes in bone marrow have also been described [167]; anemia and immune deficiency have also been observed in old age individuals. Although in the absence of other recognizable diseases, these conditions are typically mild and associated with minimal morbidity or mortality, they are magnified in the presence of a chronic disease and can lead to predisposition to infection and mortality. 

In this context, the loss of function of aging hematopoietic stem cells has been observed, as has the decline of stem cell function if other types of stem cells in the elderly, i.e., the regenerative capacity of stem cells declines, and this in turn contributes to the ageing process [168]. This is an important subject from the point of view of possible stem cell therapy, as well as other types of therapeutic interventions in the elderly. Stem cell-based treatments appear as a promising cure in age-related diseases, such as cardiovascular and neurodegenerative diseases [57], although it has been proven that the efficacy of autologous stem cell therapies is likely to decline with increasing donor age. Therefore, it has been proposed that molecules that target specific age-related pathways may restore the function of aged stem cells, being proposed that NF-κB, which is over-activated in aged tissues, may represent a molecular target for enhancing the regenerative potential of aged stem cells. Herein we exemplify recent studies which show that the rejuvenation of aged stem cell function is feasible (Table 5). Among them, it has been shown that muscle-derived stem/progenitor cells (MDSPCs) function is impaired with age [169], while, on the contrary, MDSPC-based therapies with young cells allow tissue repair following muscle, bone, cartilage, and cardiac injury [170,171]. These authors [172], using *Rela* haploinsufficient (*Rela^+/−^*) aged mice, have demonstrated that pharmacologic or genetic inhibition of NF-κB activation increases myogenic differentiation, while aged stem cells in which NF-κB activation is not inhibited cannot provide the muscle cells necessary to repair damaged tissue, suggesting that the aged phenotype of muscle stem cells may be reversible, and that the pharmacologic targeting of the NF-κB pathway may enhance the efficacy of cell-based therapies, which will be very useful in the case of an aged patient population. In another study, using transgenic mice expressing a constitutively active form of *Ikkβ* in muscle stem cells, a link between stem cell functional exhaustion and NF-κB-dependent telomere shortening was established in disease-related chronic injuries such as in Duchenne muscular dystrophy [173], suggesting that maintaining telomere length of muscle stem cells could improve its regenerative capacity also in normal and pathological ageing, not only in DMD. Moreover, the use of transgenic mice with an inducible activated NF-κB signaling in osteoprogenitors (OP)-lineage cells in skeletally mature mice has shown reduced bone mineral density in the femurs and tibias and increased bone marrow fat, resembling human osteoporosis [107] (Table 5). Thus, the authors conclude that targeting NF-κB activity as a therapeutic strategy, modulating bone remodeling by simultaneously increasing osteogenesis, and decreasing osteoclast activity could improve bone healing and prevent aging-associated bone loss in aged patients.

## 6. Conclusions

NF-κB is a ubiquitous family of transcription factors that plays a critical role in inflammation, immunity, cell proliferation, differentiation, and survival. It is necessary for the normal functioning of cells; however, overactivation of NF-κB has detrimental effects. Thus, NF-κB signaling has been shown to increase with ageing, and also, hyperactivation of NF-κB has been related with the development and progression of almost all aged-associated diseases. On the other hand, it has also been shown that phenotypic characteristics of aged organs can be reversed by inhibiting the activation of NF-κB. Therefore, approaches based on the inhibition of the signaling of different members of NF-κB have been proposed for the treatment of diseases associated with old age. In particular, recent stem cell therapy for the treatment of certain pathologies in the elderly can be improved by reversing the decline in stem cell functions by blocking the activation of NF-κB. Nevertheless, although the ability of NF-κB to control inflammation is particularly attractive for the development of specific inhibitors of this pathway in the treatment of age-related inflammatory human diseases, and putatively offers great clinical potential, it also encompasses high risks; i.e., since NF-κB controls multiple functions in cell homeostasis—including a functional immune response, cell cycle, and cell death—disruption of normal cellular responses by inhibiting NF-κB can have adverse consequences, such as immune suppression and tissue damage. Thus, it has been shown that inhibitors of IKKα and IKKβ, which due to their critical role in the NF-κB pathway would effectively block the whole pathway, have not cleared phase 2 studies as they produce gross toxicity [174,175]. Therefore, the balance between therapeutic benefit and potential changes in normal cellular function and response during drug-induced NF-κB inhibition is one of the challenges in future clinical studies, the development of pharmacokinetic studies being mandatory to optimize both the level and duration of NF-κB inhibition in order to prevent undesirable and harmful consequences for health.

## Figures and Tables

**Table 1:** Studies related to the activation of NF-κB and the development of premature aging and aged-related cancer.

NF-κB Gene	Genetic Modification	Aging Phenotype and Proposed Mechanisms	Cancer Development
*Nfkb1^−/−^* [65]	*Nfkb1^−/−^* mice, lacking both the p105 precursor and p50 subunit	Premature ageing. Reduced regeneration in liver and gut. Chronic inflammation; ROS-mediated exacerbation of telomere dysfunction and cell senescence. Mice between 12 and 132 weeks of age were analyzed.	
*Nfkb1^−/−^* [64]	*Nfkb1^−/−^* mice,	Increase inflammation, premature cellular senescence, phospho-H2AX accumulation and lower levels of spontaneous apoptosis in brains and MEFs from *Nfkb1^−/−^*, compared to *Nfkb1*^+/+^ mice. Mice of 12 and 18 months of age were analyzed.	
*Cyld* [66]	*K5-Cyld^C/S^* mice expressing a dominant negative, catalytically inactive, form of CYLD driven by the K5 promoter.	Premature ageing of skin, pancreas, liver, stomach, and thymus. Chronic over-activation of NF-κB; systemic inflammation; VEGF and c-Myc induction. Spontaneous development of different types of tumors. Young (1- to 9-month-old) and old (20- to 24-month-old) mice were analyzed.	Lung, gastric, non-melanoma skin cancer; HCC.
*Ikkβ* [67]	*K5-Ikkβ* mice overexpressing IKKβ under the control of the K5 promoter.	Over-activation of the canonical pathway of NF-κB; chronic inflammation in oral epithelia. Unpublished data: skin atrophy, loss of hair follicles and fat and sebaceous gland hyperplasia at early age (6 months). Mice between 3 and 20 months of age were analyzed.	Spontaneous tumors in oral epithelia.
*Rela^−/−^* [46]	*Sirt6/rela^+/−^* mice. Derived from *Sirt6-*deficient mice crossed with *Rela^+/−^* mice.	Deficiency of mammalian *SIRT6* leads to shortened lifespan and an ageing-like phenotype. SIRT6 interacts with p65 attenuating NF-κB signaling. Multiple *Sirt6*-deficient tissues show increased activity NF-κB -driven gene expression programs. Haploinsufficiency of *rela* rescues the early lethality and degenerative syndrome of *Sirt6*-deficient mice. 21- to 29-day-old mice were analyzed.	
*Rela^−/−^* [74]	A model of XFE progeroid syndrome, *Ercc1^–/^*^Δ^ mice; murine *Ercc1^–/–^* mice: deficient in DNA excision repair protein ERCC-1. Crosses with *Rela^+/–^* mice.	*Ercc1^–/^*^Δ^ mice present a progeroid phenotype. Genetic depletion of one allele of the *Rela* gene or treatment with a pharmacological inhibitor of IKK, delayed the age-related symptoms and pathologies in *Ercc1^–/^*^Δ^ mice such as trembling, kyphosis, and sarcopenia, as well as oxidative DNA damage and stress, and cellular senescence. Mice of 21 days of age, as well as 3- and 24-month-old mice were studied.	
*Rela^−/−^* [68]	*Zmspte24^−/−^Rela^+/−^* mice derived from *Zmpste24-*deficient mice crossed with *rela^+/−^* mice.	Progeroid syndrome: Progeroid laminopathy by defects of the nuclear lamina, phenocopying most features of HGPS (Gilford Progeria Syndrome). *Zmpste24^−/−^* mice accumulate farnesylated prelamin A at the nuclear envelope, leading to activation of ATM kinase, which cooperates with nuclear IKKγ. This results in the activation of NF-κB and up-regulation of proinflammatory cytokines. Chronic inflammatory state affects distant cells. *Zmpste24^−/−^Rela^+/−^* mice showed recovery of skin and immunological alterations and display a retardation in the ageing process and extended longevity. Three-month-old mice were analyzed.	
*Ikkβ* [53]	*MIKK* mice with muscle-specific expression of constitutively active *Ikkβ^SS177/181EE^* (muscle creatine kinase promoter, *MCK*). MISR mice: expression of *IkBα ^SS32/36AA^* (superrepressor) in muscle that inactivates NF-κB.	*MIKK* mice shows constitutive activation of NF-κB and undergo decreased skeletal muscle mass and body weight; reduced contractile function in muscles. *MIKK x MISR* mice reversed muscle atrophy. IKKβ/NF-κB activation causes accelerated protein breakdown through ubiquitin-dependent proteolysis mediated by E3ligase MuRF1. Pharmacologic NF-κB inhibition in *MIKK* mice reversed the muscle atrophy. Mice between one week and six months of age were analyzed.	
*Ikkβ,Rela* [76]	Ablation of 1 allele of *Rela* in a *mdx* mouse model of *DMD* (*dystrophin*-deficient mice) and conditional deletion of *Ikkβ* in these mice.	*DMD* mice resembles a premature aging syndrome: their myogenic stem cells become prematurely senescent. The deletion of 1 allele of *rela* improved the pathology of dystrophic muscles in mdx mice. *Ikkβ* deletion both in the immune compartment and skeletal muscle fibers reduces inflammation and necrosis through the reduction of NF-κB activity and promotes regeneration in dystrophic muscles. Mice between 4- to -12 weeks of age were analyzed.	
*Ikkβ* [77]	N/*Ikkβ*lox/lox mice: *Ikkβ*lox/lox mice crossed with *Nestin-Cre* mice.	N/*Ikkβ*lox/lox mice showed a pronounced phenotype of longevity, with median lifespan 23% longer. The longevity phenotype of this genetic model could be a result of IKKβ inhibition in neurons and glia. IKK-β and NF-κB repress gonadotropin-releasing hormone (GnRH) to mediate aging-related hypothalamic GnRH decline associated to systemic ageing. Mice aged 6 to 26 weeks were studied.	

**Table 2:** Mouse models of the NF-κB signaling pathway resembling ageing-associated human diseases.

NF-κB Gene	Genetic Modification	Recapitulated Disease and Proposed Mechanisms
		Eye Diseases
*Ikkβ* [87]	Specific knock-out of *Ikkβ* in astroglia. Use of the cre/loxP system under the regulation of the mouse glial *Gfap* to generate the *GFAP*-*ikkβ* mice.	Glaucoma *GFAP*-*ikkβ* mice lacking *Ikkβ* in astroglia show a high decrease in ocular hypertension and impeded inflammatory and neurodegenerative outcomes in the retina and optic nerve. This is the result of the inhibition of astroglial NF-κB activation, and decreased levels of proinflammatory cytokines in astroglia. Two- to three-month-old mice were analyzed.
*Rela* [89]	Generation of knock-in mice expressing the p65 S276D phosphomimetic protein, (*p65^PD^* mice), which show hyperactivation of NF-κB. Crossing of *p65^PD^* mice with mice deficient in *Tnf receptor 1*) to obtain the *p65^PD^/Tnfrsf1a^−/−^* mice.	Dry eye disease *p65^PD^* mice show a systemic hyperinflammatory condition due to increased TNF-α production, and died within 20 d after birth (5- and 10-day-old mice were studied). Crossing with *Tnfr1* knockout mice rescues the phenotype, but aged p*65^PD^/Tnfrsf1a^−/−^* mice (3.5 to 8 months of age mice were analyzed) develop chronic keratitis or keratoconjunctivitis sicca (DED), with severe corneal epithelial lesions, marked neovascularization and inflammation of the corneal stroma, and increased levels of TNF-α, IL-1β, and MMP-9. DED chronic keratitis is independent of TNFR1 signaling but dependent on NF-κB.
		**Neurodegenerative Diseases**
*c-Rel* [93]	*c-Rel* gene null mutation generated by inserting the neomycin cassette into the fifth exon of the *c-Rel* gene.	Parkinson’s disease *c-Rel* KO mice develop PD-like neuropathology with ageing, manifested by age-dependent locomotor deficits reminiscent of bradykinesia and muscle rigidity. Loss of dopaminergic neurons, increased levels of iron and DMT1, and accumulation of aggregated α-synuclein with activation of microglial cells An altered balance between p65- and c-REL-mediated effects on neurons is proposed. Mice aged 2- to 18-month-old were analyzed.
*c-Rel* [92]	*c-Rel* gene null mutation generated by inserting the neomycin cassette into the fifth exon of the *c-Rel* gene.	Parkinson’s disease In addition to the phenotype described ([80]), *c-Rel^−/−^* mice show symptoms and pathology peculiar of prodromal syndrome and a Braak-like stereotyped diffusion of synucleinopathy mimicking sporadic Parkinson´s disease. Potential involvement of changes in levels of proteins controlling mitochondrial homeostasis, ROS generation and scavenging are proposed. Mice from 2 to 18 months of age were analyzed.
*Iκbα* [98]	NcKO mice: mice with CNS-specific *Iκbα* deletion (by crossingi*Iκbα* flox with Nestin-Cre mice); CcKO and GcKO mice: mice with selective *Iκbα* deletion in neurons or in astrocytes, respectively (by crossing *IkBa* flox with *CaMKIIa-Cre* mice; or with GFAP-Cre mice).	Alzheimer´s disease This model links Amyloid β with synaptic defects and neuronal hyperexcitability though the sustained NF-κB activity in astrocytes, which results in the release of the complement protein C3 and the disruption of the dendritic morphology and network function characteristic of AD. Dysregulation of neuron-glia interaction through NF-κB/C3/C3aR signaling contribute to synaptic dysfunction in AD. Mice from 2 to 18 months of age were studied.
*Iκbα* [101]	Neuron-specific expression of *IkBα* super-repressor (*IκBα*-SR) driven by *NFH* neurofilament H promoter. Crosses of *IκB*-SR mice with familial ALS-linked mutant *Tdp-43* (G348C and A315T) and *Sod1* (G93A) mice.	Amyotrophic lateral sclerosis Mitigation of ALS symptoms and extension of life span in double transgenic mice *IκBα-SR/TDP-43* and *IκBα-SR/SOD1G93A* mice. The inhibition of NF-κB activity by the expression of *IκBα*-SR in neurons alleviates TDP-43 in neuropathology through an induction of autophagy. Reduction of misfolded SOD1 levels in and *IκBα-SR/SOD1G93A* mice. Mice from 4 to 16 months of age were studied.
		**Osteoporosis**
*Ikkβ* [107]	Osteoprogenitor (OP)-specific, doxycycline-regulated *Ikkβ*-activated mouse model (iNF-κB/OP). These mice have activated NF-κB signaling in OP-lineage cells upon DOX withdrawal in skeletally mature mice.	iNF-κB/OP mice, with increased NF-κB activation in OP-lineage cells, showed reduced bone mineral density in the femurs and tibias and increased bone marrow fat, resembling human osteoporosis. The mechanisms involved are the decreased expression of osteogenic markers (Runx2 and osteocalcin) and the increased adipogenic markers (PPAR-γ and C/EBP) in mesenchymal stem cells (MSCs) of iNF-κB/OP mice. Mice of 12 and 36 weeks of age were analyzed.
		**Osteoarthritis**
*Ikkα* [111]	Aggrecan-driven cartilage-specific *Ikkα* KO mice in chondrocytes. cKO mice are treated with tamoxifen for the specific induction.	Wilt type (WT) and cKO underwent destabilization of the medial meniscus (DMM) surgery to induce OA. cKO mice are protected from cartilage degradation after surgical DMM. cKO mice showed both reduced cartilage degradation and collagenase activity. Mice from 12 to 24 weeks of age were studied.
		**Muscle Diseases**
*Nfκb1* [116]	*Nfκb1^−^*^/−^ and *bcl3^−^*^/−^ mice.	Mice with KO of *Nfκb1* or *Bcl3* gene are resistant to muscle protein loss and functional deficit due to muscular disuse; they exhibit inhibition of fiber atrophy. Activation of the canonical pathway of NF-κB by TNF-α is associated with muscle protein loss.
*Ikbα, c-Rel* [118]	Expression of a dominant negative *IκBα* (*IκBαΔN*) in soleus muscles of rats (unloaded or weight bearing).*c-Rel^−^*^/−^ mice are also used.	IκBαΔN expression in soleus muscles inhibits NF-κB activation and abolishes the unloading-induced increase in both NF-κB activation and total ubiquitinated protein, resulting in an inhibition of 40% fiber atrophy. The expression of genes upregulated in muscle atrophy is also inhibited (*MAFbx/Atrogin-1*, *Nedd4*, *IEX*, *4E-BP1*, *FOXO3a*, *Cathepsin L*).
*Ikkβ* [117]	*IKKβ^mk^*^o^ mice exhibit muscle-specific NF-κB inhibition, through targeted deletion of *Ikkβ*. Muscle atrophy was induced by sciatic nerve denervation.	^o^ mice, lacking NF-κB activation in muscle cells showed improved skeletal muscle strength and shifted muscle fiber distribution. In response to denervation, *Ikkβ* depletion protected against atrophy, maintaining fiber type, size, and strength, increasing protein synthesis (through the activation of protein synthetic pathways: AKT, GSK3α/β, mTOR, and p70S6K), and decreasing protein degradation (muscle RING finger 1 (MuRF1). Additionally, *IKKβ^mk^*^o^ mice showed muscle regeneration after damage by enhanced satellite cell activation and reduced fibrosis.
*Ikkβ* [115]	Mice with specific expression of a constitutive kinase-active *Ikkβ* in *Pax7*-expressing satellite cells (inducible activation by Tamoxifen).	The selective activation of IKKβ in Pax7+ cells of transgenic mice led to an aggravated decline in muscle mass and fiber size. Overexpression of Pax7, induced by serum factors from cachectic mice in an NF-κB–dependent manner, was sufficient to induce atrophy in normal muscle, while under tumor conditions, the reduction of Pax7 or exogenous addition of its downstream target, MyoD, reversed wasting by restoring cell differentiation and fusion with injured fibers.

**Table 3:** Mouse models of the NF-κB signaling pathway resembling ageing-associated heart diseases.

NF-κB Gene	Genetic Modification	Age-Related Disease
		Heart Diseases
*Nfkb1* [130]	*Nfkb1-*KO mice. Chronic infusion of angiotensin II, increases systemic blood pressure and pro-inflammatory cytokines in the myocardium and provokes ventricular hypertrophy.	Cardiac hypertrophy *Nfkb1*-deficiency attenuates myocardial inflammation and hypertrophy in response to chronic infusion of angiotensin II without deteriorating cardiac function. Blockade NF-κB activation leads to abrogation of JNK phosphorylation in response to chronic infusion of angiotensin II. Mice from 8- to 12-week-old were analyzed.
*Nfkb1* [133]	*Nfkb1-*KO mice. Cross of *Nfkb1-*KO mice with *TNF-*TG mice (with cardiac-specific overexpression of TNF-α) as a model of cytokine-induced cardiomyopathy.	Cardiac dysfunction and remodeling Blockade of NF-κB in *Nfkb1*-KO mice reverses cardiac dysfunction and remodeling; improves cardiac function and survival without affecting inflammation in TNF-α-induced cardiomyopathy; it also blocks MMP-9 activation. Neonatal to 12-week-old mice were studied.
*Nfkb1* [132]	*Nfkb1*-deficient mice. Myocardial infarction was induced by ligation of the left coronary artery.	Myocardial infarction Targeted disruption of *Nfkb1* reduces ventricular rupture, and improves cardiac function and survival after myocardial infarction (MI) with amelioration of myocyte hypertrophy and interstitial fibrosis. Mechanisms proposed are: chronic inhibition of NF-κB; amelioration of myocyte hypertrophy and interstitial fibrosis; selective abrogation of JNK phosphorylation; lower infarction area. Mice from 8- to 26-week-old were analyzed.
*Nfkb1* [131]	*Nfkb1*-deficient mice. Myocardial infarction was induced by ligation of the left coronary artery.	Myocardial infarction Absence of the NF-κBp50 subunit improves early survival and reduces left ventricular dilatation after myocardial infarction. Absence of the NF-κBp50 subunit reduces collagen content and MMP-9 expression after myocardial infarction (which are involved in progressive left ventricular remodeling). Mice from 8- to 24 weeks of age were analyzed.
*Ikkβ, Iκbα, Nemo* [134]	*Tet O.IKKβ-CA* mice. Inducible transgenic mice with cardiomyocyte-specific expression of constitutively active IKKβ. *IκBα-3M*; *IKKβ-DN*, mice with a floxed *Nemo* allele. For induction of viral myocarditis, mice were infected intraperitoneally with purified CVB3.	Cardiomyopathy and heart failure In adult animals, IKKβ activation led to inflammatory dilated cardiomyopathy and heart failure. Upon transgene inactivation, the disease was reversed, even at an advanced stage. In vivo expression of the IκBα superrepressor prevented the development of the disease. Constitutively active IKKβ expression induces myocyte atrophy; excessive inflammatory response with enhanced levels of inflammatory cytokines, and an IFN type I signature, with activation of the *IFN-stimulated gene 15* (*ISG15*) pathway. In cardiomyocytes lacking *Nemo*, the induction of *ISG15* was attenuated. Mice from 4- to 24-week-old were studied.
*Ikbα* [135]	*Tg-IκBα^S32A,S36A^* mice with myocyte-specific expression of a transdominant mutant human *IκBα* (*α-MyHC* promoter) HF induced by surgery.	Heart failure *Tg-IκBα^S32A,S36A^* mice exhibit improved post-infarction survival and alleviated LV remodeling, They do not show pro-inflammatory cytokine expression or fibrosis. Display decreased apoptosis and stimulated adaptive endoplasmic reticulum stress responses in HF systolic function. Myocyte *NF-κB* abrogation induces a marked decrease of both NF-κB p65 activation and cytokine expression. Mice from 10- to 24-week-old were analyzed.
*Rela* [136]	Cardiac-specific deletion of *Rela* using a Cre-loxP system.	Cardiac hypertrophy and remodeling *Rela* deficiency in the heart of mouse decreases the hypertrophic response after pressure overload stimulation, and the degree of pathological remodeling, while preserves contractile function. Transcriptional regulatory mechanism whereby NF-κB and NFAT directly interact and synergistically promote transcriptional activation in cardiomyocytes. 8- and 12-week-old mice were studied.
		**Atherosclerosis**
*Nfkb1* [139]	*Nfkb1^−/−^* mice, subjected to chronic intermittent hypoxia (CIH) and high cholesterol diet (HCD).	*Nfkb1* gene deletion diminished CIH + HCD-induced NF-κB activation and abolished CIH + HCD induced atherosclerosis. *Nfkb1* gene deletion through the blockage of NF-κB activation inhibits CIH + HCD-induced activation of three major atherogenic mechanisms: vascular wall inflammation, hypercholesterolemia (p50 reduces serum cholesterol level), and macrophage foam cell formation. Mice between 7- and 42.6-week-old were analyzed.
*Nfkb1* [140]	*Apoe-*KO mice and mice deficient in *Nfkb1* gene *(p50-KO*) were crossed and obtained *ApoE-p50-DKO* mice.	CIH downregulates hepatic low-density lipoprotein receptor and HMG-CoA reductase expression in *ApoE-p50-DKO* but not in *ApoE-KO* mice, showing the protective role of p50 in CIH-induced atherosclerosis by inhibiting CIH-induced inflammation and hypercholesterolemia. 7- to 37-week-old were analyzed.
*Ikkγ, IkBα* [138]	Endothelium-restricted inhibition of NF-κB by ablation of *Nemo/IKKγ* or expression of dominant-negative *IkBα* in *ApoE-KO* mice: *Tie1NEMO^EC^-^KO^/ApoE^−/−^;Tie2NEMO^EC^-^KO^/ApoE^−/−^* (this latter tamoxifen-induced); and *Tie2DNIkBa/ApoE^−/−^* mice were used.	*IKKγ or IkBα* ablation in endothelial cells of *ApoE^−/−^* mice (a well-established mouse model of atherosclerosis), reduced atherosclerotic plaque formation in mice fed with a cholesterol rich diet. Inhibition of NF-κB activation abrogates adhesion molecule induction in endothelial cells, impaired macrophage recruitment to atherosclerotic plaques, and reduced expression of cytokines and chemokines in the aorta. Mice between 8- and 18-week-old were analyzed
*Ikkβ* [144]	*Ikkβ* deficiency in smooth muscle cells (SMCs) driven by a *SM22Cre-Ikkβ*-flox system. To increase susceptibility to spontaneous atherosclerotic lesion development, the *SM22Cre+IkkβF/F* mice were crossed with *LDLR^−/−^* mice.	Deficiency of *IKKβ* in SMCs rendered low density lipoprotein receptor-null mice resistant to vascular inflammation and atherosclerosis induced by high-fat feeding. *SM22Cre+IkkBF/FLDLR^−/−^* mice had decreased levels of inflammatory cytokines (IL-1β, TNF-α, and MCP-1) in the atherosclerotic lesions, arterial walls, and plasma, indicating a reduction in vascular inflammation associated with chronic inflammation. 16-week-old mice were analyzed.

**Table 4:** Age-related diseases that entail an increased risk of cancer development.

NF-κB Gene	Genetic Modification	Recapitulated Disease and Proposed Mechanisms
		Type 2 Diabetes Mellitus/Obesity/Insulin Resistance
*Ikkβ* [147]	*Ikkβ^+/−^* mice*. Ikkβ^+/+^Lep^ob/ob^* obtained by crossing *Ikkβ^+/−^* mice with *ob/ob* mice (*Lep^ob/ob^*), a mouse model for type 2 diabetes, obesity and insulin-resistance.	*Ikkβ^+/−^* mice are protected against the development of insulin resistance during high-fat feeding; they show lower concentrations of glucose and insulin in blood. *Ikkβ* heterozygosity also diminishes levels of blood glucose concentrations and improves insulin sensitivity in *Lep^ob/ob^* mice. Eight-week-old mice were analyzed.
*Ikkβ* [149]	Transgenic mice with conditional inhibition of *Ikkβ* in hepatocytes (*Ikbkb*^∆*hep*^) or myeloid cells (*Ikbkb*^∆*mye*^).	The inhibition of NF-κB pathway in myeloid cells improved insulin sensitivity in all tissues, while the inhibition of this pathway in hepatocytes maintained insulin sensitivity only in the liver: IKKβ acts locally in the liver, inducing proinflammatory mediators, which act only in a paracrine manner to downregulate insulin sensitivity in the liver, and systematically in myeloid cells. 3- to 14-month-old mice were studied.
*Ikkβ*, *Iκbα* [148]	Constitutive activation of *Ikkβ* in hepatocytes (LIKK mice). Expression of *Iκbα* supper-repressor in the liver of mice (LISK mice).	LKK mice show a type 2 diabetes phenotype, with hyperglycemia, and hepatic and systemic diet-induced insulin resistance, through mechanisms involving hepatocellular activation of NF-κB; increased levels of cytokines in liver and muscle and activation of resident Kupffer cells. LISR mice reversed the phenotype of LIKK mice. Mice between 4 and 16 weeks of age were analyzed.
*Ikkβ* [144]	*Ikkβ* deficiency in smooth muscle cells (SMCs) driven by a *SM22Cre-Ikkβ-flox* system.	*Ikkβ*-deficient mice were resistant to diet-induced obesity and metabolic disorders: lower glucose and insulin concentration in blood; reduced hepatic triglycerides and cholesterol. Mechanisms involved: inhibition of NF-κB; increased levels of the uncoupling protein-1 (UCP-1) and increased thermogenesis; lower expression of both hepatic lipogenic genes (*SREBP1c, SCD-1, and PPARγ*), and inflammatory cytokines. Mice from 8- to 28-week-old were analyzed.
*Rela* [150]	Mice *L-p65-*KO with a deletion of *Rela* gene in the liver (*p65^+/−^* mice) were generated by crossing floxed-*Rela* and *Alb-cre* mice.	*L-p65*-KO exhibited improvement hepatic insulin sensitivity, and a decrease gene expression in hepatic gluconeogenesis (feed with a high fat diet). The inhibition of the NF-κB pathway decreased the expression levels of cAMP and the phosphorylation of CREB in the liver of *L-p65-KO* mice. The low levels of cAMP results from increased expression of PDE3B, a cAMP-degrading enzyme. Mice between 8 and 32 weeks of age were studied.
*Iκbα* [151]	Mice expressing the *Iκbα* superrepressor under the *Tie2* enhancer (*E-DNIκB* mice). Crossing of *E-DNIκB* mice (a genetic model for obesity-diabetes syndrome), generates the *E-DNIkB; A^y^/+* mice.	Functionally inhibition of NF-κB specifically in endothelial cells prevents obesity-related metabolic deteriorations, with a decrease both in the macrophage infiltration into adipose tissue, and plasma oxidative stress markers induced by obesity; it also prevents age-related insulin resistance and vascular senescence at the time that extents the life span. Mice aged 8 to 50 weeks were analyzed.
*Nfkb1* [153]	*Nfkb1*-KO mice.	*Nfkb1*-deficient mice have improved insulin sensitivity. They exhibit lower expression levels of the p70 ribosomal protein S6 kinase (p70s6k or S6K1) in the liver, and reduced activity of IKKβ and IKKγ. Mice from 6- to 26-week-old were analyzed.
*Nfkb1* [154]	*Nfkb1*-KO mice.	*Nfkb1*-KO mice had both higher fatty acid utilization and oxidation in the liver, and, when feeding with a high-fat diet, they are resistant to fat accumulation and adipose tissue inflammation. Mice between 1 and 23 weeks of age were studied.
*Nfkb1*[155]	*Nfkb1*-KO mice.	*Nfkb1*-KO mice exhibits reduced body weight gain on a high-fat diet, reduced plasma triglyceride levels and adiposity. The reduced susceptibility to diet-induced obesity and dyslipidemia in p50-deficient mice results from an increase in metabolic rate, associated with elevated skeletal muscle oxidative metabolism and decreased DGAT2 expression. Mice between 10 and 34 weeks of age were analyzed.
		**Liver Diseases**
*Nfkb1* [160]	*Nfkb1*-deficient mice. *Nfkb1^1S340A/S340A^* mice carrying a mutation designed to selectively disrupt p50:p50:HDAC1 complexes.	Chronic liver disease Loss of *Nfkb1* promotes ageing associated chronic liver disease (CLD) (steatosis, neutrophilia, fibrosis, hepatocyte telomere damage, dysplasia) and HCC development. Hepatic lesions in aged *Nfkb1-/-* mice were associated with highly elevated numbers of hepatic neutrophils, which stimulates hepatocellular ROS and telomere DNA damage. Mice between 4 weeks and 20 months of age were studied.
*Nfkb1, Rela* [156]	*Nfkb1*-KO and *Rela*-KO mice.	Hepatic steatosis Hepatic steatosis was decreased in *Nfkb1-*KO mice; also, they showed reduction of both lipogenic genes (*scd1, Fas and Cd36*) and lipogenic proteins (SREBP1c and SCD1). Implication of a signaling pathway of *Nfkb1-/HDAC1/SCREBP1c* in hepatocytes in the control of hepatic steatosis. Inactivation of NF-κB p65 did not alter the hepatic steatosis. Mice between 8 and 28 weeks of age were studied.
*Cyld* [66]	K5-*Cyld^C/S^* mice. Expression of a mutant *Cyld* gene carrying a point mutation C/S that acts as a dominant negative in keratin 5 expressing cells.	Chronic hepatitis K5-*Cyld^C/S^* exhibit signs of premature aged liver, generalized inflammation, with multifocal chronic hepatitis; develop spontaneous HCC and hepatocellular adenomas. Chronic NF-κBp65 activation. Tumors likely develop as a consequence both of the premature aging and the systemic inflammation of the transgenic mice. Young (1- to 9-month-old) and old (20- to 24-month-old) mice were analyzed.
*Nik, Ikkα* [165]	Hepatocyte-specific *Nik* (*Nik*Dhep) and *Ikkα* (*ikkα*Dhep) knockout mice.	Progression of liver disease Pathological activation of hepatic NIK or IKKα likely blocks hepatocyte replication, contributing to liver disease progression in PHx mouse models. The NIK/IKK*α* pathway suppresses reparative hepatocyte proliferation at least in part by inhibiting the JAK2/STAT3 pathway. Mice 8 and 18 weeks of age were studied.

**Table 5:** Improvement of the decline of stem cell functions in ageing individuals by NF-κB blockade.

NF-κB Gene	Genetic Modification	Involvement of NF-κB in the Ageing of Progenitor Cells. Proposed Mechanisms
		Muscle-derived stem/progenitor cells
*Rela* [172]	*Rela* haploinsufficient (aged *p65^+/−^*) mice.	Aged *p65^+/−^* MDSPCs retained myogenic potential in vitro and had a higher resistance to oxidative stress-induced cell death. Genetic inhibition of NF-κB activation increases myogenic differentiation and improves resistance to oxidative stress. 14-day-old and 24-month-old mice were studied.
*Ikkβ, nemo* [173]	*IkkβCA^MuSC^* mice express a constitutively active (CA) form of the *Ikkβ* gene in MuSCs. *NemoKO^MuSC^*: Mice deficient in *Nemo* subunit in MuSCs. *Mdx* mice: Dystrophin mutant mouse.	Persistent activation of NF-κB in *IkkβCA^MuSC^* mice leads to telomere shortening and impairment of regeneration; in *Mdx/IkkβCA^MuSC^* mice it exacerbates the progression of dystrophy. The mechanism involves Ku80 dysregulation (a protein that binds to telomeres, aid the localization of other shelterins, and regulate telomere length) and increased DNA damage, specifically on telomeres, resulting in MuSC loss and, subsequently, skeletal muscle regenerative failure. Mice between 2- and 12 months of age were analyzed.
		**Osteoprogenitors**
*Ikkβ* [107]	*iNf-κb/OP* mice: express a constitutively active mutant *IKKβ* in osteoprogenitor (OP)-lineage cells, upon Dox withdrawal in skeletally mature mice.	*iNf-κb/OP* mice showed reduced bone mineral density in the femur and tibia, and increased bone marrow fat, resembling human osteoporosis. Decreased expression levels of osteogenic marker (Runx2 and osteocalcin) and increased adipogenic marker (PPAR-γ and C/EBP) in mesenchymal stem cells (MSCs) of *iNf-κb/OP* mice. Mice of 12 and 36 weeks of age were studied.

## Abbreviations

AD: Alzheimer disease; ALS: amyotrophic lateral sclerosis; ASH: alcoholic steatohepatitis; CIH: chronic intermittent hypoxia; CLD: chronic liver disease; CNS: Central Nervous System; DED: dry eye disease; DMD: Duchenne muscular dystrophy, d.n.: dominant negative; Dox: doxycycline; HCC: hepatocarcinomas; HCD: high cholesterol diet; HDAC1: histone deacetylase 1; HGPS: Gilford Progeria Syndrome; HT: heart failure; LDLR: low density lipoprotein receptor-deficient mice; LV: left ventricular remodeling; MDSPCs: muscle-derived stem/progenitor cells; MEFs: mouse embryonic fibroblasts; MI: myocardial infarction; mTOR: mammalian target of rapamycin; MuSC: muscle stem cell; NASH: non-alcoholic steatohepatitis; NFAT: nuclear factors of activated T cells; NF-kB: nuclear factor kappa-light-chain-enhancer of activated B cells; NFTs: neurofibrillary tangles; IK: NF-κB-inducing kinase; OA: Osteoarthritis; OP: osteoporosis progenitors; PD: Parkinson´s Disease; RGC: retinal ganglion cell; ROS: reactive oxygen species; SASP: senescence-associated secretory phenotype; SGNs: spiral ganglion neurons; SMCs: smooth muscle cells; T2D: type 2 diabetes mellitus; TNF: tumor necrosis factor.
